# Ant Diversity and Distribution along Elevation Gradients in the Australian Wet Tropics: The Importance of Seasonal Moisture Stability

**DOI:** 10.1371/journal.pone.0153420

**Published:** 2016-04-13

**Authors:** Somayeh Nowrouzi, Alan N. Andersen, Sarina Macfadyen, Kyran M. Staunton, Jeremy VanDerWal, Simon K. A. Robson

**Affiliations:** 1 Centre for Tropical Biodiversity & Climate Change, College of Marine and Ecosystem Sciences, James Cook University, Townsville, QLD 4811, Australia; 2 CSIRO Land & Water Flagship, Darwin, NT 0822, Australia; 3 CSIRO Land & Water Flagship, Canberra, ACT 2601, Australia; 4 eResearch Centre, Division of Research and Innovation, James Cook University, Townsville, QLD 4811, Australia; Arizona State University, UNITED STATES

## Abstract

The threat of anthropogenic climate change has seen a renewed focus on understanding contemporary patterns of species distribution. This is especially the case for the biota of tropical mountains, because tropical species often have particularly narrow elevational ranges and there are high levels of short-range endemism. Here we describe geographic patterns of ant diversity and distribution in the World Heritage-listed rainforests of the Australian Wet Tropics (AWT), revealing seasonal moisture stability to be an important environmental correlate of elevational patterns of species composition. We sampled ants in leaf litter, on the litter surface and on tree trunks at 26 sites from six subregions spanning five degrees of latitude and elevation ranges from 100–1,300 m. A total of 296 species from 63 genera were recorded. Species richness showed a slight peak at mid elevations, and did not vary significantly with latitude. Species composition varied substantially between subregions, and many species have highly localised distributions. There was very marked species turnover with elevation, with a particularly striking compositional disjunction between 600 m and 800 m at each subregion. This disjunction coincides with a strong environmental threshold of seasonal stability in moisture associated with cloud ‘stripping’. Our study therefore provides further support for climatic stability as a potential mechanism underlying patterns of diversity. The average height of orographic cloud layers is predicted to rise under global warming, and associated shifts in seasonal moisture stability may exacerbate biotic change caused by rising temperature alone.

## Introduction

Concerns over the impacts of climate change on biodiversity [1–5] have created an urgent imperative for understanding patterns and drivers of species distributions. Our understanding of species distributions is especially limited for invertebrates, which constitute the great majority of species and play dominant roles in energy and nutrient flow in most terrestrial ecosystems [6–8]. Invertebrates are also likely to be particularly powerful indicators of biodiversity responses to climate change, because of their high sensitivity to temperature and rainfall, and short generation times [9].

There is particular concern about the impact of climate change on the biota of tropical mountains. Even though tropical areas are predicted to warm at lower rates than temperate regions, the response to warming may be greater in tropical assemblages [10, 11]. Compared with temperate regions, tropical species often have narrower elevational ranges because of greater climatic change with elevation and narrower thermal tolerances [12, 13]. Tropical species with narrow elevational ranges are likely to be highly sensitive to climate change and high-elevation species are especially vulnerable to a warming climate because of a lack of dispersal options [14–16].

Tropical biodiversity is strongly influenced by climatic stability [17]. Regions with stable climates allow the evolution of finer specialisations and adaptations than do areas with variable climates [18, 19]. As such, climatically stable areas tend to have high species richness and many range-restricted species [20]. Moisture stability is a key component of climatic stability in tropical regions [21], and has important consequences for biodiversity and ecosystem function [21, 22]. Moisture from persistent clouds can provide montane rainforests with moisture stability by buffering against rainfall seasonality [23, 24]. In a process known as ‘cloud-stripping’, fog droplets collecting on vegetation can account for the majority of the water input to rainforests during the dry season months [25, 26]. This enhanced moisture availability is an important factor influencing species’ distributions [27, 28].

Historical climatic stability is a major driver of biodiversity patterns in the World-Heritage-listed rainforests of the Australian Wet Tropics (AWT), which occur in association with a coastal chain of mountains ranging up to 1,600 m elevation. The extent of rainforest in the AWT has undergone marked climate-induced contractions and expansions over the past 20,000 years, with only limited areas supporting rainforest throughout this period [29]. These stable areas have acted as biodiversity refugia [30] during drier climates and are contemporary centres of diversity and endemism [31, 32]. The distributions of rainforest species in the AWT are also strongly influenced by the extent of seasonal stability in moisture availability. Rainfall seasonality is a major driver of latitudinal and longitudinal variation in vegetation structure and composition in the AWT [33], and is believed to significantly affect bird richness due to dry-season depletions of food resources [34]. The orographic cloud layer sits at about 600 m elevation [35, 36], and seasonal moisture stability above this level is significantly higher than below [26]. This has important implications for river flows and availability of habitat for plant and animal species that rely on moist conditions [37, 38]. However, the extent to which the orographic cloud layer drives patterns of biodiversity has been poorly documented.

This paper describes rainforest ant diversity and distribution in the AWT, with a particular focus on the association between the orographic cloud layer and elevational patterns of diversity and composition. Ants are an ideal focal taxon for studying species distributions. They are a dominant faunal group in tropical rainforests [39–42] and are highly sensitive to climatic variation. Temperature is a dominant factor influencing ant distributions globally [13, 43, 44]. Within the tropics, ant diversity generally declines with increasing latitude [44, 45] and at higher elevations [46, 47]. This is consistent with metabolic theory that sees energy as a global driver of biological diversity [48–50]. However, tropical ant diversity can be highest at mid rather than low elevations [51], as is often the case in other climatic regions [52–54]. This can be explained by the mid-domain effect, where mid elevations are overlap zones for both lowland and highland taxa [55]. Ants also display high levels of species turnover with variation in elevation [28, 46, 52, 56, 57] indicating that their distributions are likely to be highly sensitive to global temperature change [58–61].

The overall objective of our study is to describe variation in ant diversity and composition with elevation across the full latitudinal range of rainforests in the AWT. We particularly focus on the hypothesis that there is a marked elevational shift in species composition associated with the orographic cloud layer.

## Methods

### Study system

**Fig 1:**
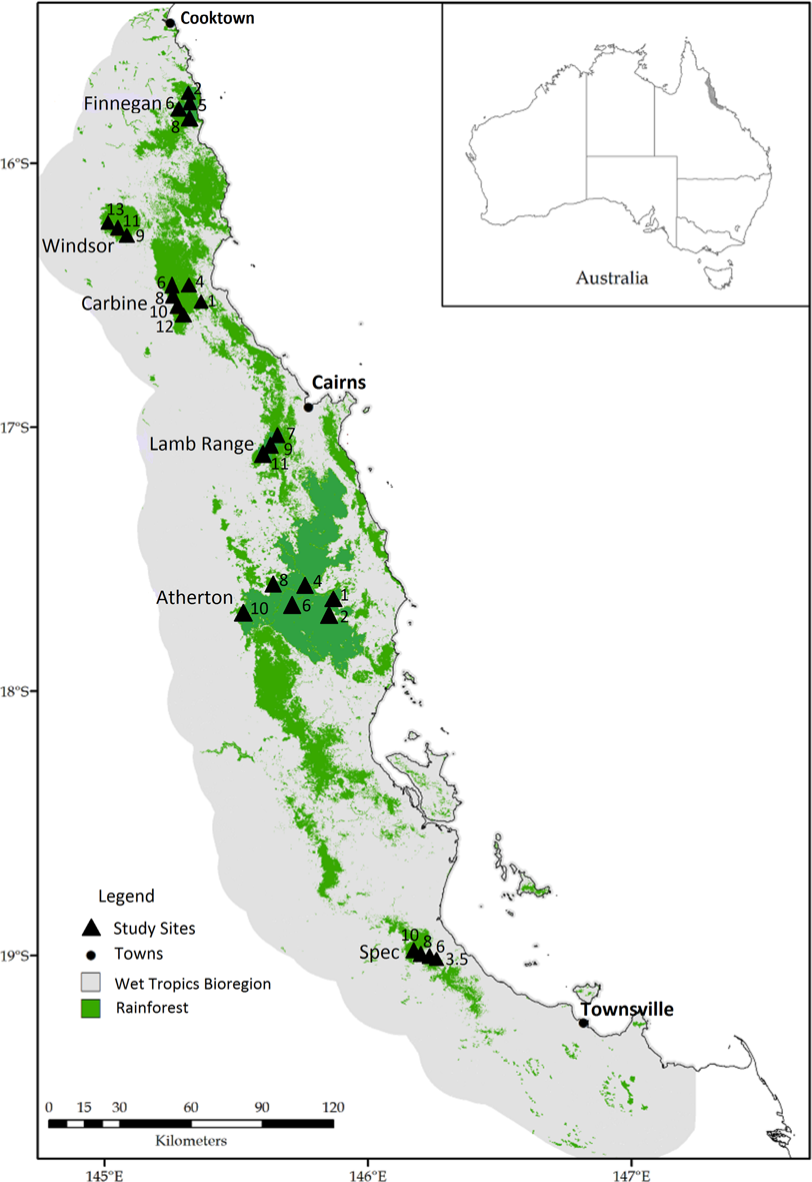
Map showing the current extent of rainforest (green shading) in the Australian Wet Tropics bioregion (grey shading), with locations of sampling sites indicated by triangles, with numbers representing elevation (‘00 m a.s.l.). Reprinted from [15] under a CC BY license, with permission from Kyran M. Staunton, original copyright 2014.

Mean annual rainfall in the AWT varies from about 1,500 to 9,000 mm, with 75–90% occurring between November and April [62]. Mean temperature declines at a rate of about 1°C for every 200 m increase in elevation [63]. Vegetation in the AWT is dominated by sclerophyll woodlands and open forests, but includes approximately 10,000 km^2^ of rainforests, mostly at higher elevation [64] (Fig 1, S1 Appendix). Despite their relatively small area, the rainforests are recognised as a major biodiversity hotspot of global significance due to their extraordinary biological richness and biogeographical uniqueness [65].

The rainforest ant fauna of the AWT has very strong South-East Asian affinities, and contrasts sharply with that of adjacent open sclerophyll habitats, which are dominated by autocthonous, arid-adapted taxa [66–68]. The AWT fauna includes several South-East Asian-based genera that occur nowhere else in Australia, as well as many others whose distributions elsewhere in Australia are restricted to rainforest patches further south along the eastern coast or west in the monsoonal zone [69, 70]. A large proportion of the species remain undescribed; for example, a recent revision of the predominantly rainforest genus *Myrmecina* increased the number of described Australian species from 2 to 13 [71].

### Study sites

Sampling was conducted at 26 long-term sites (Fig 1) established by the Centre for Tropical Biodiversity and Climate Change at James Cook University to cover the full latitudinal and elevational range of rainforest in the AWT [72]. The sites were distributed across six subregions, ranging from the Finnegan subregion near Cooktown in the north, to Mt Spec near Townsville in the south over a distance of approximately 500 km. This covers the full latitudinal range of Australia’s wet tropical rainforests. All sites were located on granite-derived soils except for those in the Atherton subregion where more-fertile basaltic soils are present [37, 73]. The elevational range of study sites varied among subregions, due to differences in the availability and accessibility of rainforest habitats. Most lowland rainforest in the AWT has been cleared for agriculture, and no lowland sites were available from Mt Windsor or Lamb Range. At Mt Spec, rainforest does not naturally occur below 300 m. Similarly, the different subregions varied markedly in maximum elevation, ranging from 800 m at Mt Finnegan to 1,300 m at Mt Windsor.

The AWT had recently experienced two severe cyclones, cyclone Larry in 2006 [74] and cyclone Yasi in 2011 [75], both of which caused major damage, especially in lowland rainforest [76]. At Atherton, sites at 100 and 200 m showed severe cyclone damage, with many broken trunks and fallen trees. A high abundance of the Pioneer Stinging Tree (*Dendrocnide excelsa*) indicated recent, but less severe, cyclone damage at 400 and 600 m.

### Ethics statement

Ant samples were collected under Permit no. WITK11729912 from the Queensland Government Department of Environment and Heritage Protection.

### Sampling

Ants were sampled at 26 sites distributed along six elevational transects: Finnegan (four sites); Windsor, (three sites); Carbine (six sites); Lamb Range (three sites); Atherton (six sites); and Mt Spec (four sites) (Fig 1). Sites were spaced by 200 m elevation along each transect. At each site, sampling was conducted at six plots separated by 200 m along a transect that followed the elevation contour. Only three plots were located at each of the 350 m site at Mt Spec and 100 m site at Atherton due to limited rainforest cover.

Rainforest ants are highly stratified vertically, with distinct faunas associated with litter (cryptic species), the litter surface (epigaeic species) and trees (arboreal species) [42]. Therefore, comprehensive sampling requires a combination of techniques that target these different components of the fauna [77, 78]. We used bait traps to sample epigaeic and arboreal species, and litter extraction to sample cryptic species. At each plot, 10 bait traps were set on the ground along a line with 5 m intervals, and 10 were set on the closest trees at a height of about 1.5 m. Bait traps were small (1 cm in diameter, 5 cm in length) plastic vials containing a piece of canned tuna. They were set early in the morning, and collected 2 hrs later. We acknowledge that such bait trapping is likely to provide a very limited representation of the specialist arboreal fauna that occurs primarily in the canopy. Leaf litter was collected from two 0.25 m^2^ quadrats, one at each end of the 50 m transect at each plot. Samples were sieved to remove large litter fragments, and ants were extracted using Winkler Sacs over a 48-hr period. At Windsor, Carbine, Atherton and Spec, sampling was conducted on three occasions: during two wet seasons (between November and January) of 2011/12 and 2012/3, and one dry season (June to September) of 2012. At Finnegan and Lamb Range, sampling occurred only during the 2011/12 wet season. Additionally, ants were sorted from pitfall samples collected during April 2009 in a previous study across the four main subregions, Windsor, Carbine, Atherton and Spec [15]. Pitfall traps were plastic containers, 11.5 cm in diameter and 10 cm depth, protected from rain by a square metal lid (length 26 cm) fixed with wire to a ring of aviary mesh (height 7.8 cm and mesh size 2.5 cm) [79]. Three pitfall traps with 15 m spacing were established at every second plot and operated for a month.

For each sampling period, each site had 60 ground bait traps, 60 arboreal bait traps and 12 litter samples, giving a total of 132 samples. The 350 m site at Mt Spec and 100 m site at Atherton were exceptions, with half these numbers of samples. Each site from Windsor, Carbine, Atherton and Spec subregions also had 9 pitfall samples.

### Analyses

All ants were sorted to species and where possible named through comparison with identified specimens held in the CSIRO Tropical Ecosystems Research Centre (TERC) in Darwin. Unidentified species were assigned species codes that apply only to this study and highly diverse genera were identified to species group following Andersen (2000). A full set of voucher specimens are deposited in the TERC collection and a duplicate set at the James Cook University ant collection.

We assessed sampling efficiency by creating individual-based rarefaction curves, which plot the number of species against a given number of individuals taken randomly from the observed data [80, 81]. We then assessed observed species richness as proportion of the Chao 1 estimated total species richness for each subregion. Variation in observed species richness among subregions was tested by one-way ANOVA. We first analysed variation among all six subregions based on the 2011/12 wet season samples. We then analysed variation among sites at Windsor, Carbine, Atherton and Spec based on pooled data across the four sampling periods. One-way ANOVA followed by Tukey tests for post-hoc pairwise comparisons, were also performed on plot-level data to test for differences in species richness among elevational sites within each subregion. Variation in ant species composition among elevations was explored with non-metric multi-dimensional scaling (NMDS) based on Bray-Curtis dissimilarity and using species frequency of occurrence (i.e. the number of samples in which a species occurred, regardless of abundance within a sample) in the 2011/12 wet season samples. ANOSIM [82] was used to test for differences among sampling strata and subregions. We used cluster analysis (agglomerative clustering) to identify major disjunctions in compositional turnover with elevation.

Analyses were conducted using the R statistical program v3.1.0 packages: the *adehabitat* package for Chao 1 richness estimation and ANOVA analyses, *iNEXT* package for rarefaction curves and the *vegan* package for NMDS and ANOSIM analyses [83, 84].

## Results

### Faunal overview

A total of 79,853 individual ants were collected, belonging to 296 species from 63 genera (S2 Appendix). The genera with highest numbers of species were *Pheidole* (40 species), *Strumigenys* (22), *Anonychomyrma* (15) and *Rhytidoponera* (14). The most abundant genera were *Pheidole* (33% of total species records) and *Anonychomyrma* (19%). The most abundant species overall were *Anonychomyrma gilberti* (10% of total species records) and *Pheidole* sp. A2 (*ampla* gp.; 6%) (S2 Appendix). The fauna included three introduced species: *Monomorium floricola* (recorded from all subregions), *Tetramorium bicarinatum* (three subregions) and *T*. *simillimum* (single record only).

**Fig 2:**
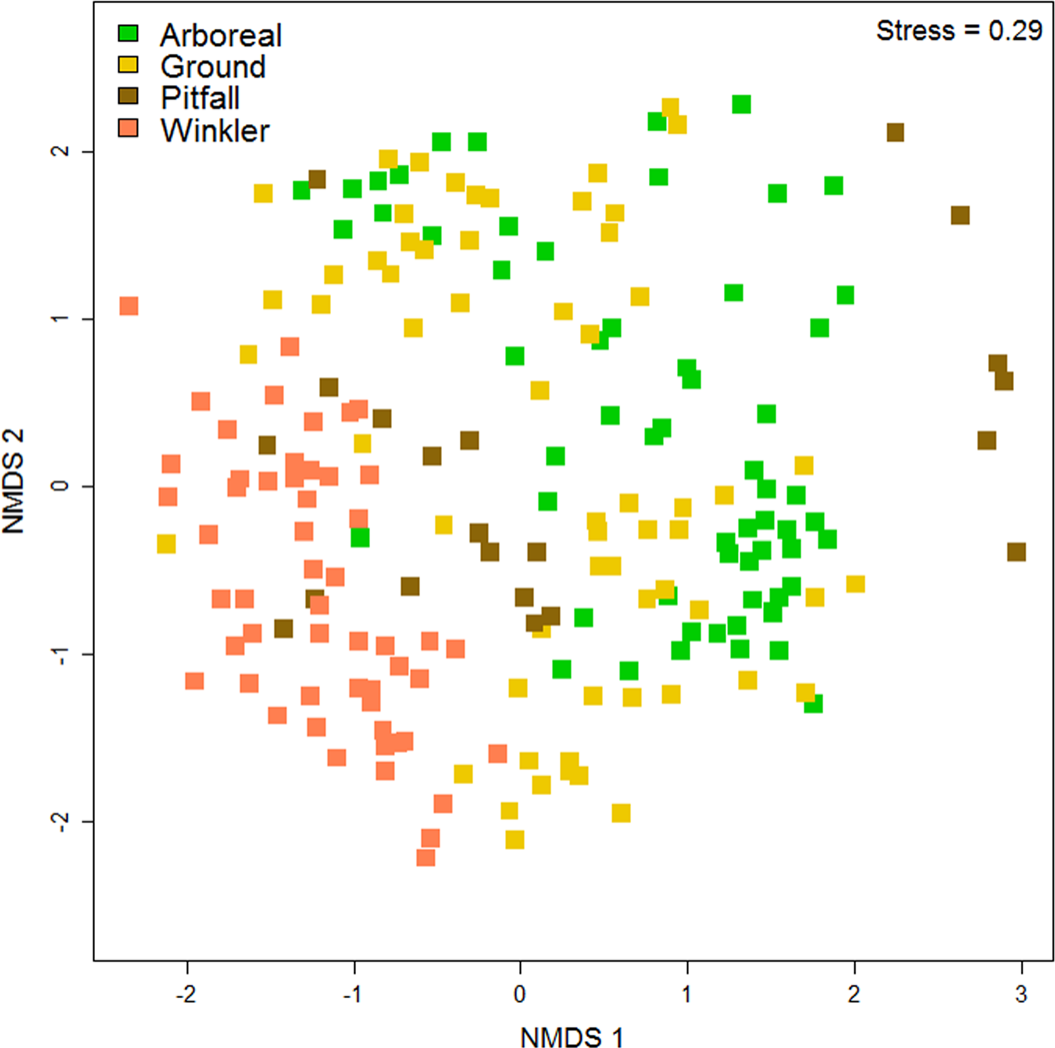
NMDS of samples from different techniques, based on species frequencies of occurrence. Each point represents a sample from one site.

Ant species composition varied with sampling method. This variation was systematically related to habitat stratum, following a gradient from Winkler samples to arboreal baits in ordination space (Fig 2). Most pitfall samples were positioned between those from Winklers and arboreal baits following the gradient of habitat strata. However, there was a cluster of 6 pitfall samples that formed a clear outlier which can be related to the very low numbers of species recorded in them. These outlier sites were from Atherton, which had a mean of only five species per site compared to 25 species at other subregions. ANOSIM showed that species composition in Winkler samples was significantly (P<0.01) different to those from all other methods and that samples from ground and arboreal baits were also significantly different (P<0.01).

Only nine (3%) species occurred in all six subregions: *Rhytidoponera* nr. *victoriae*, *Nylanderia glabrior*, *Carebara* sp. A, *Monomorium floricola*, *Pheidole* sp. A13 (*ampla* gp.), *Solenopsis* sp. A and *Hypoponera* spp. A, B and C. Considering only the 140 native species that occurred in at least three plots (in order to limit sampling artefacts that would arise from consideration of very rare species), 26 (18.6%) were recorded in only one subregion (Table 1).

**Table 1:** Ant species occurring in at least three plots that were recorded from single subregions.

Species	Subregions from North to South					
	Finnegan	Windsor	Carbine	Lamb Range	Atherton	Spec
*Heteroponera* sp. I (*relicta* gp.)	**✓**					
*Anonychomyrma* sp. D (*biconvexa* gp.)		**✓**				
*Myrmecina inaequala *		**✓**				
*Leptogenys anitae*			**✓**			
*Leptomyrmex dolichoscapus*			**✓**			
*Lordomyrma* sp. B (*punctiventris* gp.)			**✓**			
*Myrmecina alpina*			**✓**			
*Pheidole* sp. J2 (Group J)			**✓**			
*Pheidole* sp. Q1 (*quadricuspis* gp.)			**✓**			
*Plagiolepis* sp. A			**✓**			
*Technomyrmex shattucki*			**✓**			
*Heteroponera* sp. I (*relicta* gp.)				**✓**		
*Onychomyrmex* sp. E				**✓**		
*Pheidole* sp. V9 (*variabilis* gp.)				**✓**		
*Strumigenys* sp. D (*godeffroyi* gp.)				**✓**		
*Anochetus* sp. A (*graeffei* gp.)					**✓**	
*Anonychomyrma* sp. O (nitidiceps gp.)					**✓**	
*Pheidole* sp. K (Group K)					**✓**	
*Rhytidoponera* nr. *scaberrima*					**✓**	
*Anonychomyrma* sp. M (biconvexa gp.)						**✓**
*Calyptomyrmex* sp. A						**✓**
*Carebara* sp. M						**✓**
*Heteroponera* sp. K (*relicta* gp.)						**✓**
*Hypoponera* sp. O						**✓**
*Leptogenys mjobergi*						**✓**

### Species richness

**Fig 3:**
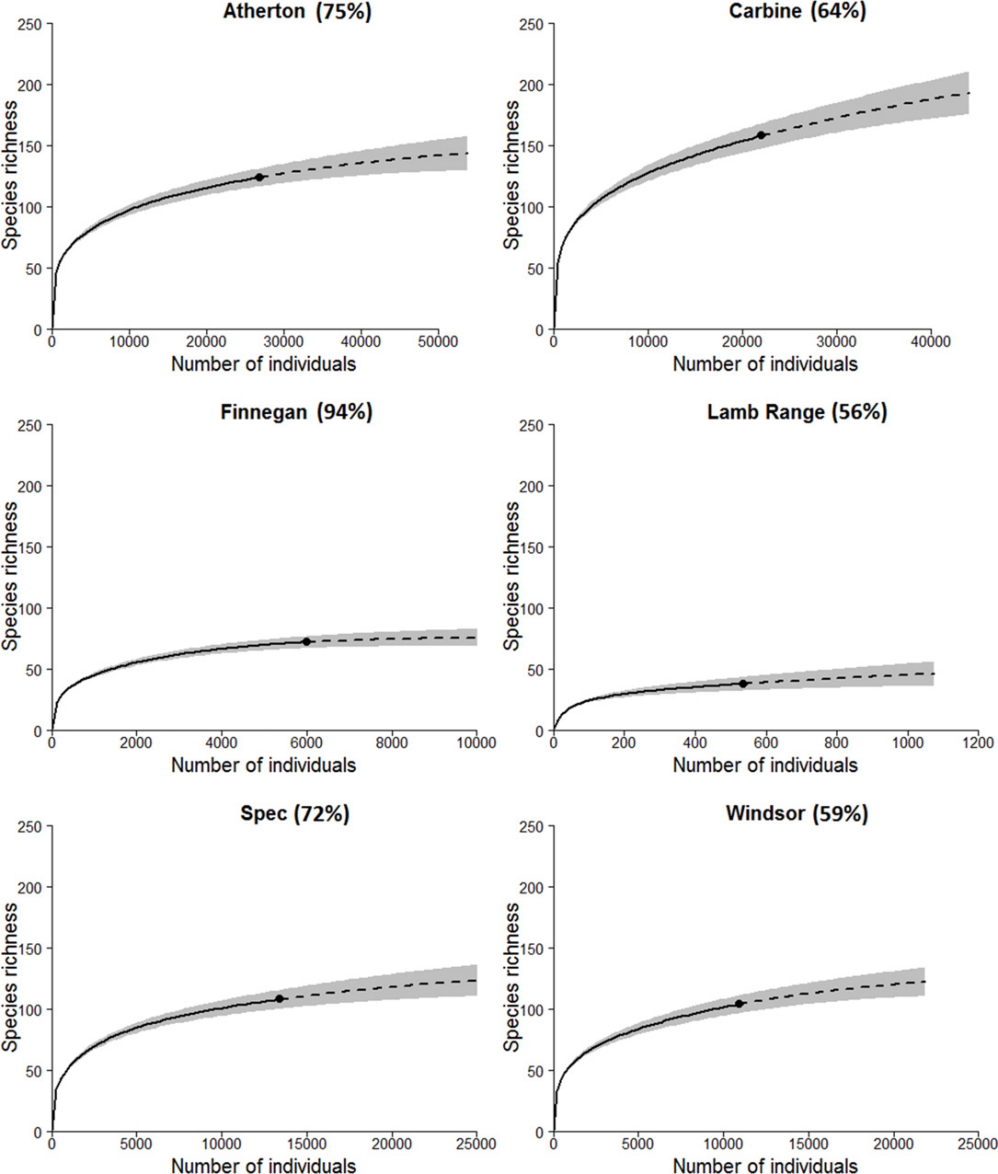
Individual-based rarefaction curves (solid) and extrapolation of the curves (dashed) in each of the six subregions based on pooled data across all sampling periods (including the 2009 pitfall trapping) for Spec, Atherton, Carbine and Windsor, and from the 2011/2012 wet season only for Finnegan and Lamb Range. Numbers represent the percentage of observed species richness as proportions of Chao 1 estimated richness. The grey shading represents 95% confidence intervals.

**Fig 4:**
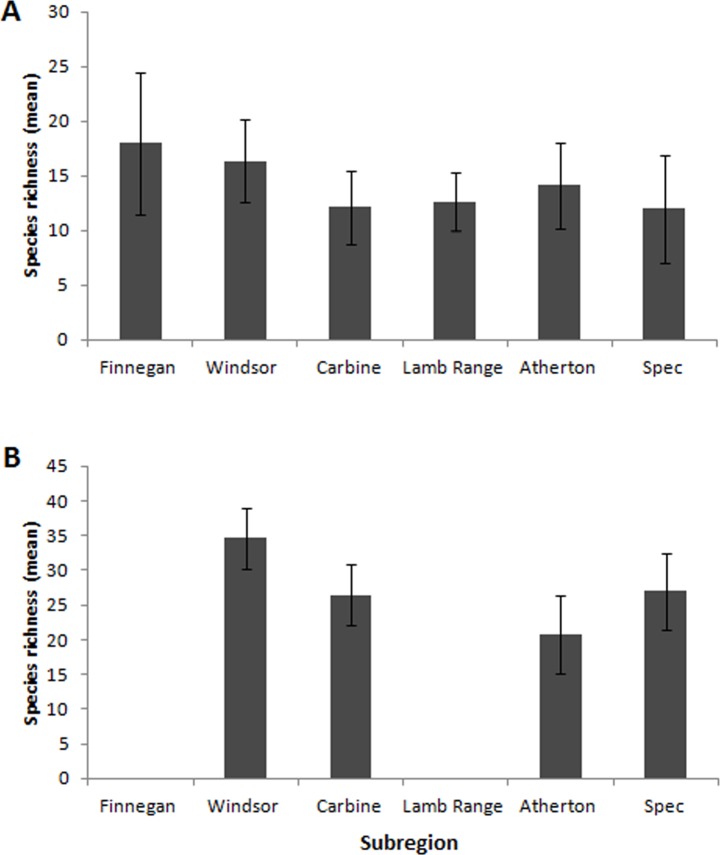
Mean site species richness at (**A**) all six subregions, using 2011–2012 wet season data, and (**B**) each of the four main subregions, based on data pooled across all sampling periods. Error bars represent 95% confidence intervals. In both cases there were no statistically significant differences between subregions.

**Fig 5:**
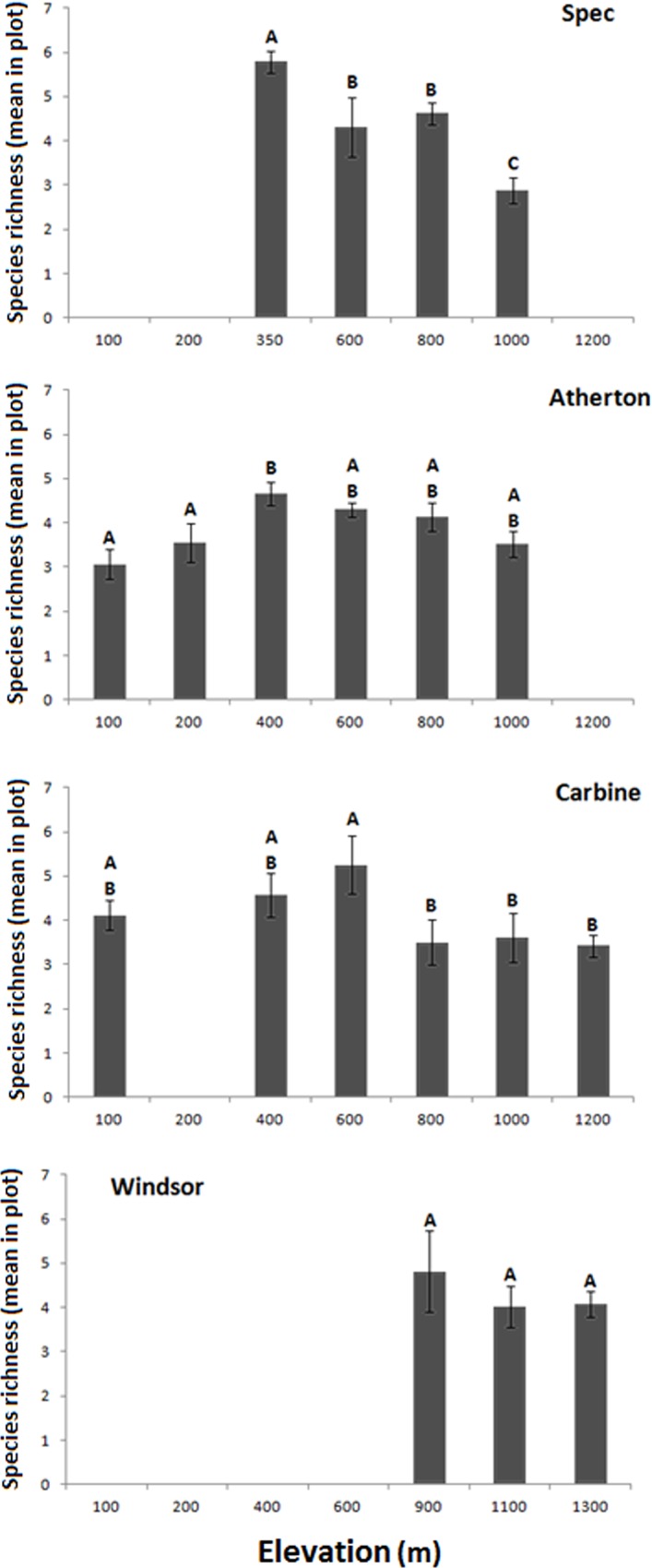
Variation in mean plot richness across elevational gradients at Windsor, Carbine, Atherton and Spec, based on data pooled across all sampling periods. Different letters indicate significant differences between elevations within a subregion.

Rarefaction curves indicate that most species occurring in each subregion were recorded, with observed species richness as a proportion of Chao 1 estimated richness ranging from 59% at Windsor to 94% at Finnegan (Fig 3). Comparisons of total richness are confounded by variable numbers of sites and sampling periods. There was a weak negative correlation between mean site richness in the 2011/12 wet season samples and latitude (R^2^ = 0.618, N = 6, P = 0.066), but ANOVA revealed no significant differences between subregions (Fig 4A; F_(5, 8)_ = 1.463, P = 0.248). There were no significant differences in mean site richness based on pooled data across sampling periods for the four subregions sampled on four occasions (Fig 4B; F_(3, 6)_ = 1.836, P = 0.184). The most extensive elevational gradients were at Carbine and Atherton, and both showed a slight peak in mean plot richness at mid elevations (Fig 5). Plot richness declined from mid to high elevation at Spec and there was no significant variation in plot richness at the three high-elevation sites at Windsor. There was no interaction between latitude and elevation (S3 Appendix).

### Species composition

**Fig 6:**
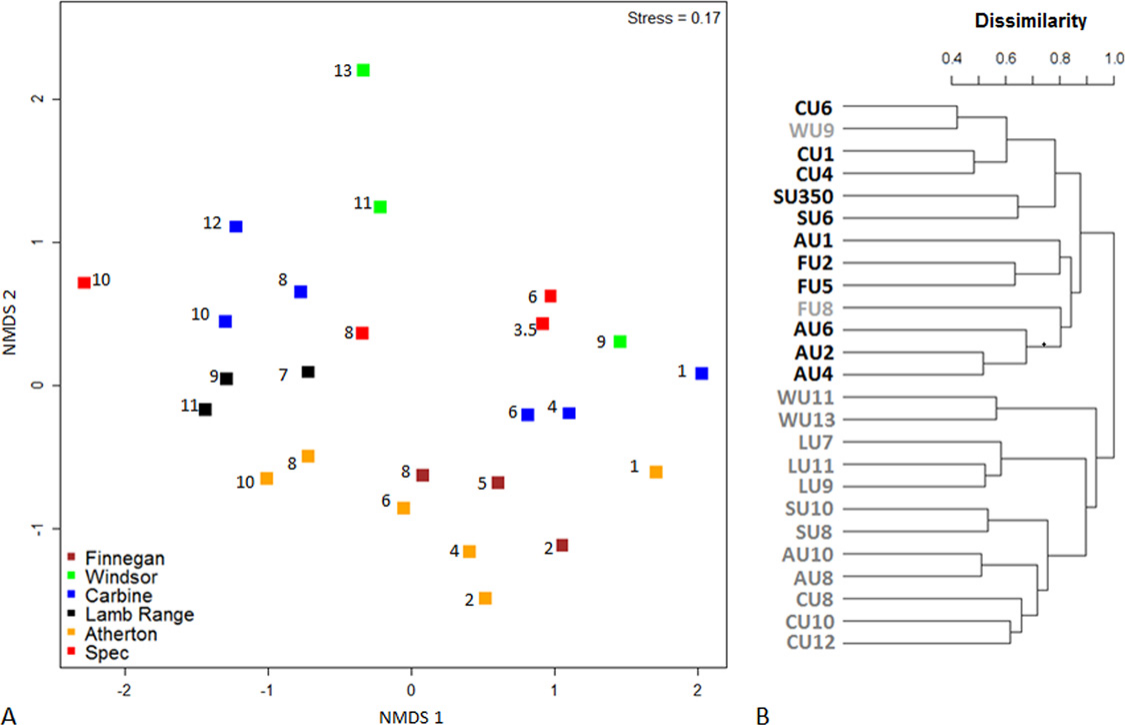
**(A)** NMDS of ant community composition captured from all 26 sites, based on species frequency data from 2011/12 wet season samples. Numbers next to each point represent elevation (‘00 m a.s.l.). (**B)** Cluster dendrogram based on dissimilarity in species composition, using the same data; sites at 600 m or lower elevation are shown in bold.

**Fig 7:**
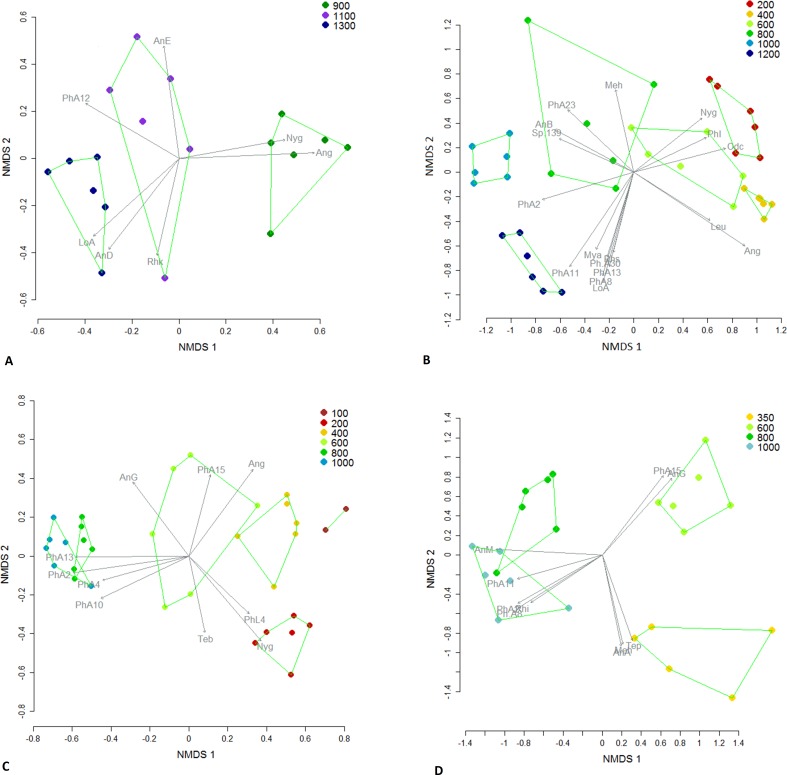
**A-D.** NMDS of ant community composition in plots at the four main subregions, based on species frequencies from pooled data across all four sampling periods. Significantly different (P<0.01) communities are shown by convex hulls. Significantly correlated (P<0.01) species are represented by vectors, with vector length proportional to level of significance. Species codes are: Ang = *Anonychomyrma gilberti*, AnA = *Anonychomyrma* sp. A *nr*. *gilberti*, AnB = *Anonychomyrma* sp. B (*biconvexa* gp.), AnD = *Anonychomyrma* sp. D (*biconvexa* gp.), AnE = *Anonychomyrma* sp. E (*nitidiceps* gp.), AnG = *Anonychomyrma* sp. G (*nitidiceps* gp.), AnM = *Anonychomyrma* sp. M, CaH = *Carebara* sp. H, CrH = *Crematogaster* sp. H, DiB = *Discothyrea* sp. B, Leu = *Leptomyrmex unicolor*, LoA = *Lordomyrma* sp. A (*punctiventris* gp.), Meh = *Meranoplus hirsutus*, MoB = *Monomorium* sp. B (*nigrius* gp.), Myn = *Myrmecia nigrocincta*, Mya = *Myrmecina alpina*, Nyg = *Nylanderia glabrior*, Odc = *Odontomachus cephalotes*, PhA1 = *Pheidole* sp.A1 (*ampla* gp.), PhA2 = *Pheidole* sp.A2 (*ampla* gp.), PhA4 = *Pheidole* sp.A4 (*ampla* gp.),PhA8 = *Pheidole* sp.A8 (*ampla* gp.), PhA10 = *Pheidole* sp.A10 (*ampla* gp.), PhA11 = *Pheidole* sp.A11 (*ampla* gp.), PhA12 = *Pheidole* sp. A12 (*ampla* gp.), PhA13 = *Pheidole* sp. A13 (*ampla* gp.), PhA15 = *Pheidole* sp.A15 (*ampla* gp.), PhA23 = *Pheidole* sp.A23 (*ampla* gp.), Ph.A30 = *Pheidole* sp.A30 (*ampla* gp.), PhI = *Pheidole* sp.I1 (*impressiceps* gp.), PhF1 = *Pheidole* sp.F1 (Group F), PhL4 = *Pheidole* sp.L4 (*longiceps* gp.), PrD = *Prolasius* sp.D, Rhs = *Rhytidoponera scaberrima*, Rhi = *Rhytidoponera impressa*, Rhk = *Rhytidoponera kurandensis*, Rhp = *Rhytidoponera purpurea*, StA = *Stigmacros* sp.A, Sty = *Strumigenys yaleopteura*, Ten = *Technomyrmex nitens*, Teb = *Tetramorium bicarinatum*, Tep = *Tetramorium pacificum*.

NMDS revealed substantial site clustering according to subregion (Fig 6A) and ANOSIM showed that many pairs of subregions had significantly dissimilar species composition (Table 2). Compositional variation was not systematic with latitude; for example, sites from Spec in the far south were compositionally most similar to those at Windsor and Carbine in the north (Fig 6A). For each subregion, plots within a site were tightly clustered in ordination and sites showed systematic variation with elevation (Fig 7A–7D). In each case, the distributions of a range of ant species were associated with different elevations. At Mt Spec, for example, *Anonychomyrma* sp. A, *Meranoplus hirsutus* and *Tetramorium pacificum* were associated with low elevation, *Anonychomyrma* sp. G and *Pheidole* sp. A15 were associated with mid elevation, and *Anonychomyrma* sp. M, *Pheidole* spp. A11, A2 and A8, and *Rhytidoponera impressa* were associated with high elevation (Fig 7D).

**Table 2:** ANOSIM results from comparisons of site species composition between each pair of subregions based on species frequency in 2011/2012 wet season samples. Numbers below the diagonal are dissimilarity indices, and those above are P values (P<0.05 in bold).

	Finnegan	Windsor	Carbine	Lamb Range	Atherton	Spec
**Finnegan**		0.094	0.332	**0.021**	0.096	**0.015**
**Windsor**	0.444		0.171	0.100	**0.037**	**0.028**
**Carbine**	0.054	0.167		0.167	0.151	0.137
**Lamb Range**	0.741	0.097	0.212		**0.012**	0.088
**Atherton**	0.242	0.414	0.091	0.796		**0.032**
**Spec**	0.475	0.630	0.175	0.364	0.361	

NMDS revealed clear elevational zonation of sites even when sites from different mountains were considered together (Fig 6A). There was a very marked compositional disjunction between 600 m and 800 m. With only two exceptions (800 m at Finnegan, and 900 m at Windsor), all sites higher than 600 m formed a cluster that was distinct from a cluster containing all lower elevation sites (Fig 6A). This disjunction was identified by cluster analysis as the primary division of sites based on ant species composition (Fig 6B). Within each of these primary clusters, most sites grouped according to subregion.

## Discussion

The AWT is a major biodiversity hotspot of global significance [65] and our study demonstrates that it supports a highly diverse ant fauna with high levels of spatial turnover. Each subregion supported a compositionally distinctive ant fauna, and a substantial proportion (19%) of species were recorded from a single subregion. Some of these species are known to occur elsewhere and so are not locally endemic; for example, *Technomyrmex shattucki* (recorded here only from Carbine) is distributed further south to the Tully region [85], *Leptogenys anitae* (likewise recorded here only from Carbine) occurs south to southeastern Queensland [86], and *Myrmecina inaequala* (recorded here only from Mt Windsor) is likewise very widely distributed [71]. However, we recorded *Leptomyrmex dolichoscapus* only at Carbine and it appears to be endemic to this area [87]. The same is true for *Myrmecina alpina* [71]. Other species of *Myrmecina* not recorded here also have very localised distributions within the AWT [71]. This is the case for virtually all other AWT ant genera that have undergone recent taxonomic revision, including *Orectognathus* [88], *Monomorium* [89], *Pristomyrmex* [90], *Anochetus* [91] and *Teratomyrmex* [92]. Such small-range endemism is known for other invertebrate taxa, including flightless insects [93], flightless ground beetles [94] and dung beetles [95] in the region.

There was high species turnover with elevation at all subregions, as appears to be typical for tropical ants [96]. However, the rates of turnover in our study are unusually high. For example, we recorded 50–65% species dissimilarity per 200 m change in elevation, which is much higher than the 20% in Malaysia [46]. Similarly, our figure of 90% species dissimilarity with a 400 m change in elevation shows higher rate than 55% over the same elevational change in Madagascar [97] and 50% over 500 m in Panama [27]. Other invertebrate taxa also have particular high rates of elevational turnover in the AWT. Dung beetles displayed up to 40% species dissimilarity for every 200 m [95], and beetles in general displayed 50% over elevational ranges of 500 m [93]. Such high rates of elevational turnover possibly reflect unusually narrow thermal tolerances (Huey et al 2012; Sunday et al 2014).

Species richness showed only weak patterns, in contrast to the high levels of species turnover. Ant species richness typically declines with increasing latitude [44, 98], as is the case for biological diversity more generally [50, 99]. We found a trend of declining ant richness with increasing latitude in the AWT, but it was not statistically significant. Temperature is a dominant factor regulating diversity in ant communities [44] and latitudinal gradients are typically gradients in mean temperature. However, although our limited latitudinal gradient spanned 4.8 degrees and 500 km, there was only a relatively slight decrease in mean annual temperature, from 21.3°C in the north (Finnegan) to 20°C in the south (Spec). Such a rate of reduction in mean annual temperature is much lower than the global average of 1°C for every 145 km change in latitude at a given elevation [100]. Latitudinal variation in diversity in the AWT is also highly confounded by variation in historic climatic stability, with marked spatial variation in the extent to which rainforest in the AWT has persisted over the past 20,000 years [20]. Such variation is an important driver of diversity and distribution for both vertebrates [65, 101] and invertebrates [93].

Previous studies of rainforest ant diversity along elevational gradients have shown either a monotonic decline with increasing elevation [28, 46], or a hump-shaped pattern featuring a mid-elevation peak [102, 103]. These are the two dominant elevational patterns for invertebrates more generally [104]. A full elevational gradient was represented at two of our subregions (Atherton and Carbine), and in both cases there was a slight peak in plot richness at mid elevations. The relatively low richness at low elevation at Atherton could possibly be attributed to cyclone damage. Cyclone Larry had a significant impact on the beetle assemblages of affected areas, increasing the proportional representation of open-habitat taxa [105]. However, treefall-gap formation appears to have little influence on litter ants [106]. Moreover, relatively low ant richness at lowland sites in the Atherton region was recorded prior to the recent cyclones [107] and so does not appear to be an artefact of cyclone impacts.

Our most noteworthy finding was a striking elevational disjunction in ant species composition across all subregions between 600 and 800 m. This faunistic disjunction corresponds with a major climate/vegetation boundary in the AWT associated with persistent orographic cloud. Rainforest vegetation changes from mesophyll vine forest in the lowlands to complex notophyll vine forest and microphyll vine fern forests on cloud-affected mountains [108]. Some vertebrate species are known to be restricted to moist mountain tops [109, 110], but the compositional disjunction we have documented for ants represents the clearest known faunistic association with cloud stripping in the region. There were two higher elevation sites that did not conform to the 600 m disjunction in ant species composition: 800 m at Finnegan and 900 m at Windsor. They are the only sites that have a westerly aspect and so are not subject to the moisture-laden, south-easterly winds that are the source of orographic cloud in the region.

Previous studies have shown that biodiversity patterns in the AWT are strongly influenced by regional variation in long-term climatic stability, which has determined the extent to which rainforest in the AWT has persisted over the past 20,000 years [20]. Stable patches have acted as biodiversity refuges and are now centres of diversity and endemism for a range of taxa [65, 101]. Rapid turnover of species composition, as a result of short-term climatic changes, may destabilize the ecosystem by loss of co-occurring species [10]. Our study is consistent with the notion that climatic stability over contemporary timescales is an important driver of ant biodiversity patterns along elevational gradients in the AWT and provides further support for the importance of climatic stability as a driver of tropical biodiversity patterns.

The average elevation of orographic cloud layers is predicted to rise throughout the world under global warming [111, 112], and in the AWT it is predicted to rise from about 600 to 900 m by 2050 [36]. Sites that are currently located at the bottom of the cloud layer are therefore likely to experience drier conditions under a future climate and this can be expected to exacerbate biotic change caused by rising temperatures alone. The 600–800 m elevation zone is therefore likely to be especially sensitive to a changing climate and represents a priority location to focus efforts for monitoring climate-change impacts in the AWT.

## Supporting Information

S1 Appendix Permission from the copyright holder to publish Fig 1. (DOCX)

S2 Appendix Ant species and their frequency of occurrence in the six subregions. (DOCX)

S3 Appendix Correlation between ant species richness and latitude, elevation and inetraction of these two factors. (DOCX)
