# Supplementary material for: Ant Diversity and Distribution along Elevation Gradients in the Australian Wet Tropics: The Importance of Seasonal Moisture Stability
Source: PLoS One. 2016 Apr 13;11(4):e0153420. doi: 10.1371/journal.pone.0153420 (PMC4830544; doi:10.1371/journal.pone.0153420)
Supplement: S2 Appendix — (DOCX) [file pone.0153420.s002.docx]

**Supporting information**

**S2 Appendix.** Ant species and their frequency of occurrence in the six subregions.

|  | **Finnegan** | **Windsor** | **Carbine** | **Lamb Range** | **Atherton** | **Spec** | **Total** |
| --- | --- | --- | --- | --- | --- | --- | --- |
| **Aenictinae** |  |  |  |  |  |  | **6** |
| *Aenictus aratus* |  |  |  |  |  | 1 | 1 |
| *A. nesiotis* |  |  | 1 |  |  |  | 1 |
| *A. prolixus* |  |  | 1 |  | 3 |  | 4 |
| **Amblyoponinae** |  |  |  |  |  |  | **47** |
| *Amblyopone australis* |  | 1 |  |  |  |  | 1 |
| *Amblyopone* sp. A |  | 1 |  |  |  |  | 1 |
| *Onychomyrmex hedleyi* | 1 | 2 | 11 | 1 | 10 |  | 25 |
| *Onychomyrmex* sp. B |  |  | 1 |  |  |  | 1 |
| *Onychomyrmex* sp. C |  | 4 | 1 |  |  |  | 5 |
| *Onychomyrmex* sp. D |  |  | 1 |  |  |  | 1 |
| *Onychomyrmex* sp. E |  |  |  | 3 |  |  | 3 |
| *Onychomyrmex* sp. F |  |  |  |  |  | 2 | 2 |
| *Prionopelta robynmae* |  |  | 1 | 6 | 1 |  | 8 |
| **Cerapachyinae** |  |  |  |  |  |  | **18** |
| *Cerapachys* nr. *adamus* |  | 2 |  |  |  |  | 2 |
| *C.* nr. *binodis* |  |  | 1 |  |  |  | 1 |
| *Cerapachys* sp. A (*turneri* gp.) |  |  | 1 |  |  |  | 1 |
| *Cerapachys* sp. B (*turneri* gp.) |  |  |  | 2 | 1 |  | 3 |
| *Cerapachys* sp. C (*turneri* gp.) |  |  |  |  | 1 |  | 1 |
| *Cerapachys* sp. D (*turneri* gp.) |  | 1 |  |  |  |  | 1 |
| *Sphinctomyrmex steinheili* |  |  | 1 |  | 2 |  | 3 |
| *Sphinctomyrmex* sp. B |  |  | 1 |  |  |  | 1 |
| *Sphinctomyrmex* sp. C | 2 |  | 1 |  |  |  | 3 |
| *Sphinctomyrmex* sp. D | 1 |  | 1 |  |  |  | 2 |
| **Dolichoderinae** |  |  |  |  |  |  | **1,080** |
| *Iridomyrmex* nr. *anceps* |  |  |  |  | 2 |  | 2 |
| *I. mayri* |  | 8 |  |  |  |  | 8 |
| *I. suchieri* |  | 7 |  |  | 1 |  | 8 |
| *Leptomyrmex dolichoscapus* |  |  | 18 |  |  |  | 18 |
| *L. ruficeps* |  | 4 | 3 |  | 4 | 2 | 13 |
| *L. unicolor* | 2 | 6 | 39 |  |  |  | 47 |
| *Tapinoma melanocephalum* | 4 |  |  |  |  |  | 4 |
| *Tapinoma* sp. A | 1 |  | 1 |  |  | 1 | 3 |
| *Technomyrmex cheesmanae* |  |  |  |  |  | 3 | 3 |
| *T. difficilis* |  |  | 3 |  | 1 | 2 | 6 |
| *T. nitens* | 3 | 13 | 11 |  | 4 | 3 | 34 |
| *T. quadricolor* | 4 |  | 5 | 3 | 6 | 8 | 26 |
| *T. shattucki* |  |  | 7 |  |  |  | 7 |
| *T. sophiae* |  |  |  |  |  | 1 | 1 |
| *Turneria bidentata* |  |  | 1 |  |  |  | 1 |
| *Anonychomyrma gilberti* | 17 | 81 | 227 |  | 108 | 48 | 481 |
| *A.* nr.*gilberti* |  |  | 23 |  |  | 56 | 79 |
| *Anonychomyrma* sp. B (*biconvexa* gp.) |  | 6 | 17 |  |  | 14 | 37 |
| *Anonychomyrma* sp. C (*biconvexa* gp.) |  | 17 | 4 |  |  |  | 21 |
| *Anonychomyrma* sp. D (*biconvexa* gp.) |  | 35 |  |  |  |  | 35 |
| *Anonychomyrma* sp. E (*nitidiceps* gp.) |  | 34 |  |  | 11 | 3 | 48 |
| *Anonychomyrma* sp. G (*nitidiceps* gp.) | 7 | 2 | 45 |  | 36 | 57 | 147 |
| *Anonychomyrma* sp. H (nitidiceps gp.) |  |  | 1 |  |  |  | 1 |
| *Anonychomyrma* sp. J (nitidiceps gp.) |  | 1 |  |  |  |  | 1 |
| *Anonychomyrma* sp. K (biconvexa gp.) |  |  |  |  |  |  | 0 |
| *Anonychomyrma* sp. L (nitidiceps gp.) | 1 |  | 1 |  |  |  | 2 |
| *Anonychomyrma* sp. M (biconvexa gp.) |  |  |  |  |  | 40 | 40 |
| *Anonychomyrma* sp. N (nitidiceps gp.) |  |  | 1 |  |  |  | 1 |
| *Anonychomyrma* sp. O (nitidiceps gp.) |  |  |  |  | 3 |  | 3 |
| *Anonychomyrma* sp. P (biconvexa gp.) |  |  |  |  |  | 3 | 3 |
| **Ectatomminae** |  |  |  |  |  |  | **574** |
| *Rhytidoponera chnoopyx* |  |  | 8 | 5 | 3 |  | 16 |
| *R. impressa* | 1 |  | 3 | 2 | 1 | 33 | 40 |
| *R. kurandensis* | 1 | 23 | 11 |  | 1 |  | 36 |
| *R. laticeps* |  |  | 1 |  | 4 | 6 | 11 |
| *R. metallica* |  |  |  |  |  | 1 | 1 |
| *R. purpurea* | 26 | 22 | 34 |  | 97 | 16 | 195 |
| *R. scaberrima* | 2 | 1 | 9 |  | 5 |  | 17 |
| *R.* nr. *scaberrima* |  |  |  |  | 3 |  | 3 |
| *R. spoliata* |  |  | 5 |  |  |  | 5 |
| *R.* nr. *victoriae* | 22 | 20 | 27 | 21 | 34 | 84 | 208 |
| *Rhytidoponera* sp. C | 2 |  |  |  | 13 | 4 | 19 |
| *Rhytidoponera* sp. F (*araneoides* gp.) |  |  | 3 |  | 4 | 9 | 16 |
| *Rhytidoponera* sp. G *(araneoides gp.)* | 3 |  |  |  | 2 |  | 5 |
| *Rhytidoponera* sp. H |  |  |  |  | 2 |  | 2 |
| **Formicinae** |  |  |  |  |  |  | **395** |
| *Camponotus confusus* | 1 |  | 3 |  | 4 |  | 8 |
| *C. thadeus* | 2 |  |  |  |  |  | 2 |
| *C. vitreus* |  |  | 1 |  | 1 |  | 2 |
| *Camponotus* sp. A (*aureopilus* gp.) |  |  | 1 |  |  |  | 1 |
| *Camponotus* sp. B (*macrocephalus* gp.) |  |  | 1 |  |  |  | 1 |
| *Camponotus* sp. D1 (*discors* gp.) |  |  |  |  |  | 1 | 1 |
| *Camponotus* sp. N1 (*novaehollandiae* gp.) |  |  | 2 |  |  |  | 2 |
| *Camponotus* sp. N2 (*novaehollandiae* gp.) |  | 4 |  |  |  | 2 | 6 |
| *Camponotus* sp. S1 (*subnitidus* gp.) |  |  |  |  |  | 1 | 1 |
| *Notostigma carazii* |  | 1 |  |  | 1 | 10 | 12 |
| *Nylanderia glabrior* | 27 | 16 | 48 | 5 | 114 | 31 | 241 |
| *Nylanderia* sp. B |  |  |  |  | 2 |  | 2 |
| *Nylanderia* sp. C |  | 1 |  |  |  |  | 1 |
| *Oecophylla smaragdina* | 8 |  |  |  | 29 |  | 37 |
| *Paraparatrechina* sp. A (*minutula* gp.) |  | 2 | 2 |  | 1 | 8 | 13 |
| *Paraparatrechina* sp. B (*minutula* gp.) |  |  | 1 |  |  |  | 1 |
| *Paraparatrechina* sp. C (*minutula* gp.) |  | 1 |  |  |  | 1 | 2 |
| *Paraparatrechina* sp. D |  | 2 | 2 |  |  |  | 4 |
| *Plagiolepis* sp. A |  |  | 3 |  |  |  | 3 |
| *Plagiolepis* sp. B |  |  | 1 |  |  |  | 1 |
| *Polyrhachis argentosa* | 2 |  |  |  |  |  | 2 |
| *P. delecta* | 1 |  |  |  |  | 1 | 2 |
| *P. flavibasis* |  | 1 |  |  |  |  | 1 |
| *P. monteithi* |  |  | 1 |  |  |  | 1 |
| *P. rufifemur* |  | 1 |  |  |  |  | 1 |
| *Prolasius* sp. B (*nitidissimus* gp.) | 1 |  |  | 7 | 4 |  | 12 |
| *Prolasius* sp. C (*nitidissimus* gp.) |  |  | 3 |  | 1 | 4 | 8 |
| *Prolasius* sp. D (*convexus* gp.) |  | 20 | 1 |  |  |  | 21 |
| *Stigmacros* sp. A (*pusilla* gp.) |  | 2 |  |  |  |  | 2 |
| *Stigmacros* sp. B (*pusilla* gp.) |  | 2 |  |  |  |  | 2 |
| *Stigmacros* sp. C (*aciculata* gp.) |  |  |  |  |  | 1 | 1 |
| *Stigmacros* sp. D (*pusilla* gp.) |  | 1 |  |  |  |  | 1 |
| **Heteroponerinae** |  |  |  |  |  |  | **103** |
| *Heteroponera* sp. A (*imbellis* gp.) | 2 | 8 | 26 |  | 5 | 5 | 46 |
| *Heteroponera* nr. *relicta* |  |  |  |  | 4 |  | 4 |
| *Heteroponera* nr. *trachypyx* |  |  | 1 |  | 1 |  | 2 |
| *Heteroponera relicta* |  | 1 | 32 |  |  |  | 33 |
| *Heteroponera rhodopygea* | 5 | 1 |  |  |  |  | 6 |
| *Heteroponera* ?*ecarinata* |  |  | 1 |  |  |  | 1 |
| *Heteroponera pendergrasti* |  |  |  | 4 |  |  | 4 |
| *Heteroponera darlingtonorum* |  |  |  |  |  | 7 | 7 |
| **Myrmeciinae** |  |  |  |  |  |  | **4** |
| *Myrmecia nigrocincta* |  | 1 |  |  | 1 | 2 | 4 |
| **Myrmicinae** |  |  |  |  |  |  | **2,613** |
| *Anillomyrma* sp. B |  |  | 1 |  |  |  | 1 |
| *Anillomyrma* sp. C |  |  | 1 |  |  |  | 1 |
| *Calyptomyrmex* sp. A |  |  |  |  |  | 6 | 6 |
| *Cardiocondyla atalanta* |  | 1 |  |  |  |  | 1 |
| *C.* nr. *wroughtoni* |  | 1 |  |  |  |  | 1 |
| *Cardiocondyla* sp. A (*emeryi* gp.) | 1 |  |  |  |  |  | 1 |
| *Carebara* sp. A | 6 | 7 | 22 | 6 | 8 | 11 | 60 |
| *Carebara* sp. C |  |  | 2 |  |  |  | 2 |
| *Carebara* sp. D |  | 1 |  |  |  | 1 | 2 |
| *Carebara* sp. E |  |  |  |  |  | 2 | 2 |
| *Carebara* sp. F |  | 2 |  |  |  |  | 2 |
| *Carebara* sp. G |  |  | 2 |  |  |  | 2 |
| *Carebara* sp. H |  | 1 | 2 |  |  |  | 3 |
| *Carebara* sp. I |  | 1 | 8 |  |  | 5 | 14 |
| *Carebara* sp. J |  |  | 2 |  |  |  | 2 |
| *Carebara* sp. L |  |  |  |  |  | 2 | 2 |
| *Carebara* sp. M |  |  |  |  |  | 3 | 3 |
| *Carebara* sp. N |  |  |  |  |  | 1 | 1 |
| *Carebara* sp. O |  |  |  |  |  | 1 | 1 |
| *Colobstruma biconvexa* |  |  |  |  |  | 2 | 2 |
| *Crematogaster* sp. A |  |  | 7 |  | 13 | 1 | 21 |
| *Crematogaster* sp. B |  |  | 1 |  |  |  | 1 |
| *Crematogaster* sp. C |  |  |  |  | 1 |  | 1 |
| *Crematogaster* sp. D |  |  | 1 |  |  |  | 1 |
| *Crematogaster* sp. E |  |  |  |  | 2 |  | 2 |
| *Crematogaster* sp. F |  |  | 1 |  |  |  | 1 |
| *Crematogaster* sp. G | 5 |  | 9 |  | 1 | 33 | 48 |
| *Crematogaster* sp. H |  |  | 4 |  | 12 | 3 | 19 |
| *Crematogaster* sp. I | 1 |  |  | 1 |  |  | 2 |
| *Eurhopalothrix australis* |  | 1 | 1 |  | 3 | 4 | 9 |
| *Lordomyrma* sp. A (*punctiventris* gp.) |  | 14 | 18 | 1 | 1 |  | 34 |
| *Lordomyrma* sp. B (*punctiventris* gp.) |  |  | 3 |  |  |  | 3 |
| *Lordomyrma* sp. E (*punctiventris* gp.) |  | 1 |  |  |  |  | 1 |
| *Lordomyrma* sp. F (*punctiventris* gp.) |  |  | 4 |  |  |  | 4 |
| *Mayriella abstinens* |  |  | 2 |  | 2 | 2 | 6 |
| *M. spinosior* | 1 |  |  |  |  |  | 1 |
| *Meranoplus beatoni* |  |  | 1 |  |  |  | 1 |
| *M. hirsutus* | 1 | 27 | 60 |  | 29 | 3 | 120 |
| *Monomorium dracula* |  |  | 1 |  |  |  | 1 |
| *M. floricola* | 4 | 2 | 4 | 1 | 5 | 6 | 22 |
| *M. petiolatum* |  |  | 1 |  | 13 |  | 14 |
| *M. pharaonis* |  |  |  |  |  | 1 | 1 |
| *M. sydneyense* |  | 1 |  |  |  |  | 1 |
| *M. turneri* |  |  |  |  | 2 |  | 2 |
| *Monomorium* sp. A (*albipes complex*) |  |  |  |  | 1 |  | 1 |
| *Monomorium* sp. B (*nigrius* gp.) | 1 | 3 |  |  | 1 |  | 5 |
| *Monomorium* sp. C (*rubriceps* gp.) |  |  | 1 |  | 95 |  | 96 |
| *Monomorium* sp. D (*albipes complex*) |  |  |  |  | 1 |  | 1 |
| *Monomorium* sp. E (nr. *petiolatum*) |  |  |  |  | 1 |  | 1 |
| *Monomorium* sp. H (*leae* gp.) | 2 | 2 |  |  |  | 3 | 7 |
| *Myrmecina alpina* |  |  | 9 |  |  |  | 9 |
| *M.* nr. *alpina* |  | 1 |  |  |  |  | 1 |
| *M. difficulta* |  | 1 | 1 |  |  |  | 2 |
| *M. inaequala* |  | 8 |  |  |  |  | 8 |
| *M. mjobergi* |  |  | 1 |  |  |  | 1 |
| *M. pumilla* |  |  |  | 1 |  |  | 1 |
| *M. silvarugosa* | 1 |  |  |  |  |  | 1 |
| *Myrmecina* sp. A | 1 |  |  |  |  |  | 1 |
| *Myrmecina* sp. B |  |  | 2 |  |  |  | 2 |
| *Opistopsis linnaei* |  |  |  |  |  | 1 | 1 |
| *Orectognathus nanus* |  |  |  |  |  | 2 | 2 |
| *O. robustus* |  | 1 | 1 |  | 9 | 2 | 13 |
| *O.*nr. *satan* |  | 1 |  |  |  |  | 1 |
| *Pheidole* sp. A1 (*ampla* gp.) |  | 21 |  |  | 1 |  | 22 |
| *Pheidole* sp. A10 (*ampla* gp.) |  |  | 27 |  | 50 | 14 | 91 |
| *Pheidole* sp. A11 (*ampla* gp.) |  | 9 | 57 |  | 1 | 7 | 74 |
| *Pheidole* sp. A12 (*ampla* gp.) |  | 85 | 23 |  |  |  | 108 |
| *Pheidole* sp. A13 (*ampla* gp.) | 1 | 15 | 61 | 18 | 121 | 21 | 237 |
| *Pheidole* sp. A14 (*ampla* gp.) |  | 6 | 4 | 2 | 7 | 7 | 26 |
| *Pheidole* sp. A15 (*ampla* gp.) | 6 | 12 | 13 |  | 12 | 6 | 49 |
| *Pheidole* sp. A16 (*ampla* gp.) |  |  | 1 |  |  |  | 1 |
| *Pheidole* sp. A2 (*ampla* gp.) |  | 5 | 116 | 9 | 110 | 64 | 304 |
| *Pheidole* sp. A20 (*ampla* gp.) | 2 | 3 |  |  |  |  | 5 |
| *Pheidole* sp. A23 (*ampla* gp.) |  |  | 24 |  |  | 7 | 31 |
| *Pheidole* sp. A24 (*ampla* gp.) |  |  |  | 3 | 4 | 2 | 9 |
| *Pheidole* sp. A25 (*ampla* gp.) |  |  | 1 | 1 | 14 | 5 | 21 |
| *Pheidole* sp. A26 (*ampla* gp.) |  |  |  |  | 3 |  | 3 |
| *Pheidole* sp. A3 (*ampla* gp.) |  |  | 9 |  |  |  | 9 |
| *Pheidole* sp. A30 (*ampla* gp.) |  | 2 | 13 |  | 1 | 3 | 19 |
| *Pheidole* sp. A4 (*ampla* gp.) |  | 2 | 7 |  | 42 | 5 | 56 |
| *Pheidole* sp. A5 (*ampla* gp.) |  | 1 | 6 | 4 | 3 |  | 14 |
| *Pheidole* sp. A6 (*ampla* gp.) |  | 1 | 7 |  |  |  | 8 |
| *Pheidole* sp. A7 (*ampla* gp.) |  | 5 | 2 |  | 3 | 3 | 13 |
| *Pheidole* sp. A8 (*ampla* gp.) |  | 4 | 55 | 4 | 3 | 65 | 131 |
| *Pheidole* sp. A9 (*ampla* gp.) |  |  |  |  | 4 | 7 | 11 |
| *Pheidole* sp. B | 1 |  |  |  |  |  | 1 |
| *Pheidole* sp. E1 (Group E) |  |  |  |  | 2 |  | 2 |
| *Pheidole* sp. F1 (Group F) | 18 | 1 | 28 |  | 12 | 3 | 62 |
| *Pheidole* sp. F2 (Group F) |  |  |  |  |  | 3 | 3 |
| *Pheidole* sp. ?*impressiceps* | 6 | 1 | 21 |  |  |  | 28 |
| *Pheidole* sp. J1 (Group J) |  |  | 1 |  |  |  | 1 |
| *Pheidole* sp. J2 (Group J) |  |  | 3 |  |  |  | 3 |
| *Pheidole* sp. K (Group K) |  |  |  |  | 4 |  | 4 |
| *Pheidole* sp. L1 (*lonifera* gp.) | 5 | 6 | 6 |  | 12 |  | 29 |
| *Pheidole* sp. L2 (*onifera* gp.) |  |  |  |  | 2 |  | 2 |
| *Pheidole* sp. L4 (*onifera* gp.) | 2 |  | 14 |  | 11 |  | 27 |
| *Pheidole* sp. L5 (*onifera* gp.) | 1 |  | 7 |  |  |  | 8 |
| *Pheidole* sp. Q1 (*quadricuspis* gp.) | 2 | 1 | 28 |  | 14 |  | 45 |
| *Pheidole* sp. V1 (*variabilis* gp.) | 2 | 12 | 23 |  | 5 | 40 | 82 |
| *Pheidole* sp. V2 (*variabilis* gp.) | 8 | 5 | 1 |  | 55 |  | 69 |
| *Pheidole* sp. V4 (*variabilis* gp.) | 21 | 12 | 12 |  | 7 |  | 52 |
| *Pheidole* sp. V6 (*variabilis* gp.) |  |  |  |  |  | 5 | 5 |
| *Pheidole* sp. V9 (*variabilis* gp.) |  |  |  | 5 |  |  | 5 |
| *Pheidologeton affinis* | 12 | 2 | 10 |  | 7 | 2 | 33 |
| *Podomyrma nr. grossestriata* |  |  | 2 |  |  |  | 2 |
| *P. mjobergi* |  |  |  |  | 1 | 1 | 2 |
| *P. muckeli* |  |  | 1 |  |  |  | 1 |
| *Podomyrma* sp. B |  |  |  |  |  | 2 | 2 |
| *Podomyrma* sp. C |  | 1 |  |  |  |  | 1 |
| *Podomyrma* sp. D |  | 1 |  |  | 2 |  | 3 |
| *Pristomyrmex foveolatus* | 1 |  | 2 |  | 1 |  | 4 |
| *Pristomyrmex nr. foveolatus* |  |  |  |  |  | 1 | 1 |
| *Pristomyrmex wilsoni* |  |  | 2 |  |  |  | 2 |
| *Rhopalothrix* sp. A |  |  |  |  |  | 1 | 1 |
| *Rhoptromyrmex* sp. A | 4 |  | 2 |  | 2 |  | 8 |
| *Solenopsis* sp. A | 1 | 22 | 7 | 1 | 3 | 3 | 37 |
| *Solenopsis* sp. B |  | 4 | 6 |  |  |  | 10 |
| *Solenopsis* sp. C | 1 |  | 1 |  |  |  | 2 |
| *Solenopsis* sp. D | 5 | 4 | 1 |  |  |  | 10 |
| *Solenopsis* sp. E |  | 6 |  |  |  | 1 | 7 |
| *Strumigenys cingatrix* | 1 | 1 | 3 | 3 |  | 3 | 11 |
| *S. deuteras* |  |  |  |  |  | 3 | 3 |
| *S.* nr. *deuteras* |  | 1 |  |  | 2 |  | 3 |
| *S. emdeni* |  |  | 1 |  |  |  | 1 |
| *S.* nr. *emmae* | 1 |  |  |  |  |  | 1 |
| *S. enanna* |  |  | 1 |  |  |  | 1 |
| *S. friedae* |  | 1 |  |  | 1 |  | 2 |
| *S. guttulata* |  |  |  |  | 1 | 1 | 2 |
| *S. harpyia* | 10 | 8 | 14 |  | 13 | 5 | 50 |
| *S.*nr. *harpyia* |  |  | 1 |  |  |  | 1 |
| *S. juxta* |  | 2 |  |  |  | 1 | 3 |
| *S.*nr. *jugis* |  |  |  |  |  | 1 | 1 |
| *S. minteras* |  |  |  |  |  | 1 | 1 |
| *S. orthanetes* |  | 8 | 4 | 1 | 1 | 1 | 15 |
| *S. paranetes* |  | 14 | 11 | 2 | 3 | 19 | 49 |
| *S. philiporum* |  |  | 3 | 1 | 3 |  | 7 |
| *S. semicompta* | 2 |  |  |  |  |  | 2 |
| *S.*nr. *snellingi* | 1 |  |  |  |  |  | 1 |
| *S. yaleopteura* |  |  |  |  | 1 |  | 1 |
| *Strumigenys* sp. A |  |  | 1 | 4 |  |  | 5 |
| *Strumigenys* sp. C (*godeffroyi* gp.) |  |  |  | 2 |  |  | 2 |
| *Strumigenys* sp. D (*godeffroyi* gp.) |  |  |  | 6 |  |  | 6 |
| *Tetramorium bicarinatum* |  |  | 5 | 4 | 13 |  | 22 |
| *T. ornatum* | 4 |  |  |  | 1 |  | 5 |
| *T. pacificum* | 2 |  | 4 |  | 3 | 10 | 19 |
| *T. simillimum* |  |  | 1 |  |  |  | 1 |
| *T. strictum* |  |  | 4 |  | 1 | 8 | 13 |
| *Tetramorium* sp. C |  |  |  |  | 1 | 1 | 2 |
| *Tetramorium* sp. F (*strictum* gp.) |  |  |  |  | 1 |  | 1 |
| *Tetramorium* sp. G (*pacificum* gp.) | 1 |  | 1 |  |  |  | 2 |
| *Tetramorium* sp. H (*pacificum* gp.) |  |  | 1 |  |  |  | 1 |
| *Vollenhovia* sp. A |  |  |  |  | 1 |  | 1 |
| *Vollenhovia* sp. B |  |  |  |  |  | 1 | 1 |
| *Vombisidris* sp.A | 1 |  |  |  |  |  | 1 |
| unidentified *Stenamminie* sp. A |  |  |  |  | 1 |  | 1 |
| **Ponerinae** |  |  |  |  |  |  | **302** |
| *Anochetus* nr. *graeffei* |  |  |  |  | 5 |  | 5 |
| *Hypoponera* sp. A | 1 | 35 | 18 | 9 | 18 | 19 | 100 |
| *Hypoponera* sp. B | 1 | 2 | 2 | 8 | 4 | 3 | 20 |
| *Hypoponera* sp. C | 3 | 13 | 5 | 7 | 2 | 2 | 32 |
| *Hypoponera* sp. G |  |  | 1 |  |  |  | 1 |
| *Hypoponera* sp. H |  | 2 |  |  |  |  | 2 |
| *Hypoponera* sp. J |  |  | 1 |  |  |  | 1 |
| *Hypoponera* sp. K |  | 2 |  |  |  |  | 2 |
| *Hypoponera* sp. L | 1 |  |  |  | 1 | 1 | 3 |
| *Hypoponera* sp. M |  |  |  |  |  | 1 | 1 |
| *Hypoponera* sp. N |  |  | 1 |  | 1 |  | 2 |
| *Hypoponera* sp. O |  |  |  |  |  | 3 | 3 |
| *Leptogenys anitae* |  |  | 3 |  |  |  | 3 |
| *L.*nr.*anitae* |  |  |  |  | 1 |  | 1 |
| *L. longensis* |  |  |  |  | 1 |  | 1 |
| *L. mjobergi* |  |  |  |  |  | 6 | 6 |
| *L.*nr.*mjobergi* |  | 2 | 5 | 1 | 2 |  | 10 |
| *L. sjostedti* |  |  | 1 |  |  | 1 | 2 |
| *Leptogenys* sp. C (*diminuta* gp.) |  |  | 1 |  |  |  | 1 |
| *Mesoponera australis* |  |  |  | 1 |  | 1 | 2 |
| *Myopias tenuis* |  |  | 1 |  | 2 |  | 3 |
| *Odontomachus cephalotes* | 8 |  | 42 |  | 8 | 2 | 60 |
| *Ponera* sp. A |  | 2 |  |  |  |  | 2 |
| *Ponera* sp. B |  | 2 |  |  |  |  | 2 |
| *Pseudoneoponera* sp. B (*oculata* gp.) |  |  |  |  | 3 |  | 3 |
| *Pseudoneoponera* sp. A (*porcata* gp.) |  |  | 2 |  | 2 |  | 4 |
| *Pseudoneoponera* sp. C (*porcata* gp) |  |  |  |  |  | 2 | 2 |
| *Brachyponera croceicornis* | 2 |  | 13 |  | 10 |  | 25 |
| *Cryptopone* sp. A |  | 2 |  |  |  |  | 2 |
| *Platythyrea* sp. A (*parallela* gp.) |  |  | 1 |  |  |  | 1 |
| **Proceratiinae** |  |  |  |  |  |  | **5** |
| *Discothyrea* sp. A |  |  |  |  | 1 |  | 1 |
| *Discothyrea* sp. B |  | 1 |  |  |  | 1 | 2 |
| *Discothyrea* sp. C |  |  | 1 |  |  |  | 1 |
| *Proceratium* sp. A |  |  | 1 |  |  |  | 1 |
