# Supplementary material for: Ant Diversity and Distribution along Elevation Gradients in the Australian Wet Tropics: The Importance of Seasonal Moisture Stability
Source: PLoS One. 2016 Apr 13;11(4):e0153420. doi: 10.1371/journal.pone.0153420 (PMC4830544; doi:10.1371/journal.pone.0153420)
Supplement: S3 Appendix — (DOCX) [file pone.0153420.s003.docx]

**Supporting information**

**S3 Appendix.** Correlation between ant species richness and latitude, elevation and inetraction of these two factors, based on pooled raw data from the six subregions.

|  | **Estimate** | **Std. Error** | **t value** | **Pr(>\|t\|)** |  |
| --- | --- | --- | --- | --- | --- |
| **(Intercept)** | 27.986 | 12.958 | 2.160 | 0.033 | * |
| **Latitude** | 0.613 | 0.748 | 0.819 | 0.415 |  |
|  |  |  |  |  |  |
|  |  |  |  |  |  |
|  | **Estimate** | **Std. Error** | **t value** | **Pr(>\|t\|)** |  |
| **(Intercept)** | 18.693 | 1.770 | 10.560 | 0.000 | *** |
| **Elevation** | -0.002 | 0.002 | -0.810 | 0.420 |  |
|  |  |  |  |  |  |
|  |  |  |  |  |  |
|  | **Estimate** | **Std. Error** | **t value** | **Pr(>\|t\|)** |  |
| **(Intercept)** | 37.241 | 40.824 | 0.912 | 0.364 |  |
| **Latitude** | 1.052 | 2.375 | 0.443 | 0.659 |  |
| **Elevation** | -0.008 | 0.051 | -0.158 | 0.875 |  |
| **Latitude : Elevation** | -0.001 | 0.003 | -0.112 | 0.911 |  |
