# Supplementary material for: Ant Diversity and Distribution along Elevation Gradients in the Australian Wet Tropics: The Importance of Seasonal Moisture Stability
Source: PLoS One. 2016 Apr 13;11(4):e0153420. doi: 10.1371/journal.pone.0153420 (PMC4830544; doi:10.1371/journal.pone.0153420)
Supplement: S1 Appendix — (DOCX) [file pone.0153420.s001.docx]

**Supporting information**

**S1 Appendix.** Permission from the copyright holder to publish Fig 1.

Re: Permission to publish Figure 1 of your paper

Kyran Staunton <staunton.k@gmail.com>

Thu 3/03/2016 9:09 AM

To:Nowrouzi, Sara (L&W, Black Mountain) <Sarah.Nowrouzi@csiro.au>;

Dear Somayeh Nowrouzi,

As the copyright holder of Figure 1 (Map of the Wet Tropics bioregion; doi:10.1371/journal.pone.0088635.g001), I give permission for you to publish this figure, under a Creative Commons Attribution Licence as requested by you.

Kind regards,

Dr Kyran Staunton.

On 1 March 2016 at 21:06, <[Sarah.Nowrouzi@csiro.au](mailto:Sarah.Nowrouzi@csiro.au)> wrote:

Dear Dr Kyran Staunton,

I request permission for the open-access journal PLOS ONE to publish Figure 1 of your paper (Map of the Wet Tropics bioregion; doi:10.1371/journal.pone.0088635.g001) under the Creative Commons Attribution License (CCAL) CC BY 4.0 (<http://creativecommons.org/licenses/by/4.0/>). Please be aware that this license allows unrestricted use and distribution, even commercially, by third parties. Please reply and provide explicit written permission to publish Figure 1 of your paper (Map of the Wet Tropics bioregion; doi:10.1371/journal.pone.0088635.g001) under a CC BY license.

Thanks very much for your time.

Kind regards,

Somayeh Nowrouzi
